# Cardiac asthma in elderly patients: incidence, clinical presentation and outcome

**DOI:** 10.1186/1471-2261-7-16

**Published:** 2007-05-14

**Authors:** Stéphane Jorge, Marie-Hélène Becquemin, Samuel Delerme, Mohamed Bennaceur, Richard Isnard, Rony Achkar, Bruno Riou, Jacques Boddaert, Patrick Ray

**Affiliations:** 1 Department of Emergency Medicine and Surgery, Centre Hospitalo-Universitaire (CHU) Pitié-Salpêtrière, Assistance-Publique Hôpitaux de Paris (AP-HP), 47–83 boulevard de l'Hopital, 75013 Paris, Université Pierre et Marie Curie-Paris 6, France; 2 Laboratory of pulmonary fonction test, Centre Hospitalo-Universitaire (CHU) Pitié-Salpêtrière, Assistance-Publique Hôpitaux de Paris (AP-HP), 47–83 boulevard de l'Hopital, 75013 Paris, Université Denis Diderot and UPRES 2397, France; 3 Department of cardiology. Centre Hospitalo-Universitaire (CHU) Pitié-Salpêtrière, Assistance-Publique Hôpitaux de Paris (AP-HP), 47–83 boulevard de l'Hopital, 75013 Paris, Université Pierre et Marie Curie-Paris 6, France

## Abstract

**Background:**

Cardiac asthma is common, but has been poorly investigated. The objective was to compare the characteristics and outcome of cardiac asthma with that of classical congestive heart failure (CHF) in elderly patients.

**Methods:**

Prospective study in an 1,800-bed teaching hospital.

**Results:**

Two hundred and twelve consecutive patients aged ≥ 65 years presenting with dyspnea due to CHF (mean age of 82 ± 8 years) were included. Findings of cardiac echocardiography and natriuretic peptides levels were used to confirm CHF. Cardiac asthma patients were defined as a patient with CHF and wheezing reported by attending physician upon admission to the emergency department. The CHF group (n = 137) and the cardiac asthma group (n = 75), differed for tobacco use (34% vs. 59%, p < 0.05), history of chronic obstructive pulmonary disease (16% vs. 47%, p < 0.05), peripheral arterial disease (10% vs. 24%, p < 0.05). Patients with cardiac asthma had a significantly lower pH (7.38 ± 0.08 vs. 7.43 ± 0.06, p < 0.05), and a higher PaCO_2 _(47 ± 15 vs. 41 ± 11 mmHg, p < 0.05) at admission. In the cardiac asthma group, patients had greater distal airway obstruction: forced expiratory volume in 1 second of 1.09 vs. 1.33 Liter (p < 0.05), and a forced expiratory flow at 25% to 75% of vital capacity of 0.76 vs. 0.99 Liter (p < 0.05). The in-hospital (23% vs. 19%) and one year mortality (48% vs. 43%) rates were similar.

**Conclusion:**

Patients with cardiac asthma represented one third of CHF in elderly patients. They were more hypercapnic and experienced more distal airway obstruction. However, outcomes were similar.

## Background

In Western countries, the population is getting older, and it is projected that there will be a doubling of individuals over the age of 65 and 80 years by 2030 [1]. It is estimated that about 10% of the population over the age of 80 years have heart failure [1]. Congestive heart failure (CHF) is the leading cause of acute dyspnea in elderly patients, with an in-hospital mortality ranging from 13 to 29 % [2-8]. CHF presenting with wheezing is termed cardiac asthma [9,10]. Although airflow obstruction in the setting of cardiogenic pulmonary edema (CPE) has long been familiar to clinicians, the mechanisms responsible for this observation remain obscure. The elevation of pulmonary or bronchial vascular pressure likely results in reflex bronchoconstriction [10-13]. Other potential causes of airway narrowing include a geometric decrease in airway size from reduced lung volume, obstruction from intraluminal edema fluid, and bronchial mucosal swelling [14-16]. Some investigators have found an increase in bronchial responsiveness to methacholine in patients with CHF [10,12]. However, there is little knowledge of the prevalence presentation, clinical characteristics and outcomes of cardiac asthma in elderly patients.

Therefore, the objectives of this study were to 1) determine the incidence of wheezing, though to reflect the occurrence of bronco-obstruction ("cardiac asthma"), during CHF, and 2) compare the characteristics (clinical and functional), and prognosis of patients with cardiac asthma to that of classical CPE (non wheezing CPE) in patients aged 65 years and over.

## Methods

### Study design and setting

A single-center prospective study was conducted between February 2001 and September 2002, in an emergency department (ED) of a 1,800 bed teaching urban hospital. This work was an ancillary analysis of an epidemiological study of acute respiratory failure in elderly patients (EPIDASA study) which has been previously described in detail elsewhere [5]. In the present study, patients with CHF were included and compared according to the presence or absence of wheezing. The study was approved by our Institutional Review Board, and waived informed consent was authorized because routine care of the patient was not modified.

### Selection of participants

The criteria for inclusion in the EPIDASA study were: age ≥ 65 years and acute dyspnea. In addition, one of the following criteria had to be fulfilled: 1) respiratory rate ≥ 25/min; 2) PaO_2 _≤ 70 mmHg; 3) PaCO_2 _≥ 45 mmHg with pH ≤ 7.35; 4) peripheral oxygen saturation (SpO_2_) ≤ 92 %. To be included in the present study, patient should have CHF as defined by experts (see below). There was no exclusion criteria in the EPIDASA study; we excluded only patients with obvious acute bronchial asthma, as defined by experts.

### Data collection and processing

For every patient, medical care instituted by physicians in charge included: medical history especially cardiac risk factors, previous cardiac and respiratory diseases, physical examination, arterial blood gas analysis (performed on a ABL 725™, Radiometer Copenhagen, Danemark), 12 lead electrocardiogram, and chest X-ray. Emergency treatments and admission were decided by the emergency physician according to normal practices and recommendations [3,15]. Creatinine levels were measured, and creatinine clearance was estimated with the Cockcroft formula. B-type natriuretic peptide (BNPTriage™, Biosite, Buc, France) and its N-terminal fraction (NT-proBNP Elecsys™ 2010 analyser, Roche Diagnostics, Meylan, France) levels were reported when available [4]. As recommended for confirming CHF in elderly patient, a transthoracic Doppler-echocardiography with emphasis on diastolic function was performed if available, and as quickly as possible during the hospitalization. Septal E/Ea ratio was used as a simple and reliable noninvasive surrogate for pulmonary capillary pressure regardless of rhythm [20]. Non systolic heart failure was defined when the ejection fraction of left ventricule was above 50%. Pulmonary function tests (PFTs) (performed on a Pulmonet III™, Gould, Cleveland, OH, USA or Easyone™, NDD Medizintechnik, Zurich, Switzerland) with flow-volume loop were performed on patients, accordingly to the orders of the physicians in charge. Patients performed at least three expiratory maneuvers, with a forceful and maximal effort, in order to obtain the best forced vital capacity (FVC) and expiratory flow/volume loop [17]. The following indices were measured: FVC (L), FVC (percentage of predicted), forced expiratory volume in one second (FEV_1_) (L), FEV_1 _(percentage of predicted), FEV_1_/FVC ratio (%), and forced midexpiratory flow rate (FEF_25–75%_) (percentage of predicted). Since measurement of lung volumes by pletysmography was rarely ordered by physicians in charge and subsequently performed, we did not report the results.

### Confirmation of congestive heart failure and COPD

The complete medical chart was reviewed, especially noting medications including loop diuretics, nitrate, bronchodilatators and other treatments. Response to diuretic or vasodilator, results of echocardiography-Doppler, or BNP and NT-proBNP levels [4,18] when available were specifically analyzed for the confirmation of CHF, by experts (two independent senior physicians specializing in either respirology, cardiology, internal medicine, intensive care, geriatrics or emergency medicine) [1,3,18-20]. Thereafter, patients with CHF as defined by experts were separated in two groups: patients with wheezing reported by the attending physician in the emergency room at admission were included in the cardiac asthma group and patients without wheezing were included in the classical CPE group.

The diagnosis of COPD was made by experts according to reports from a general practioner, respirologist or medical chart from previous admission; patients' current symptoms (chronic cough and sputum production), clinical findings (including clinical signs of distension); and radiographic findings (Chest X-ray or CT scan) of thoracic distension [21,22]. On the chest X-ray, thoracic distension was defined by the presence of one of the following radiological signs: enlargement of intercostal space, horizontalization of the ribs, or flat diaphragm. Patients with history of chronic bronchitis and signs of emphysema but without PFT were also included in the COPD group [22].

### Outcome measures

To determine the rate of readmission and long term survival, all patients were followed-up by a phone call three months and one year after discharge.

### Primary data analysis

Data are expressed as mean ± standard deviation (SD) or median (and interquartile 25–75%) for non-Gaussian variables. Comparison of two means was performed using the Student's t test, comparison of two medians using the Mann and Whitney test, and comparison of two proportions using the Fisher's exact method. A multivariable analysis was performed in order to assess predictors of cardiac asthma. We used a conservative approach and included in this test only variables with a p value <0.10 in the univariate analysis. All statistical comparisons were two-tailed and a p value of less than 0.05 was required to reject the null hypothesis. Statistical analysis was performed using computer NCSS 6.0 software (Statistical Solutions Ltd, Cork, Ireland).

## Results

Five hundred and fourteen patients were included, of which 212 patients had a final diagnosis of CHF [5]. From these, 75 (35%) presented with cardiac asthma, and 137 patients (65%) without wheezing had classical CPE. Table 1 compared the main characteristics of patients with CHF, of whom 135 (64%) were aged 80 years and above. Patients with cardiac asthma had a higher frequency of tobacco use and diagnosis of COPD. They presented with hypercapnic acidemia on blood gas analysis. The rate of suspicion of CHF by emergency physician was similar in both groups (83% in classical CPE versus 83% in the cardiac asthma group, non significant (NS)). Treatments in the emergency room were significantly different in the two groups with higher prescription of bronchodilators in the group of cardiac asthma. The chest X-ray showed thoracic distension more frequently in the cardiac asthma group (16% vs. 6%, p < 0.05), but less cardiomegaly (57% vs. 77%, p < 0.05).

The triggers of acute CHF were similar in the classical CPE group as compared to the cardiac asthma group: systolic arterial hypertension (26% vs. 37%, NS), myocardial ischemia (21% vs. 15%, NS), acute pneumonia (19% vs. 16%, NS), tachyarrhythmia (17% vs. 13%, NS), anemia (10% vs. 8%, NS).

Doppler-echocardiography was performed in 152 (72%) patients during the hospitalization. The rate of non systolic heart failure was similar in both groups (33% vs 41 %, NS). The median (interquartile 25–75%) BNP (n = 130) and NT-proBNP (n = 106) levels were similar in the cardiac asthma group as compared to that of classical CPE, 576 pg/mL (247–1,080) versus 615 pg/mL (275–1,050) and 4747 (1,164–20,458) versus 3921 pg/mL (1,788–8,603) respectively.

**Figure 1:**
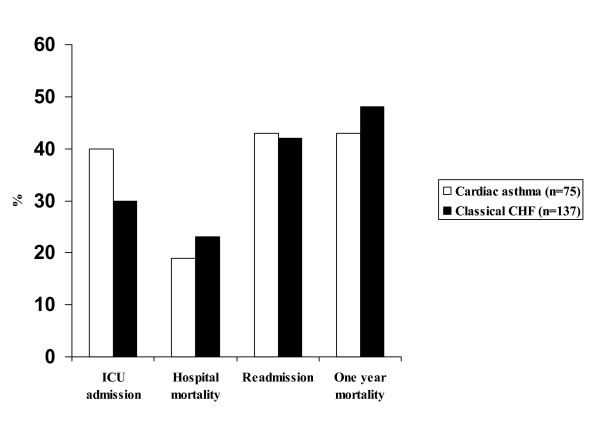
Immediate and Long Term Prognosis in Either Group.

All patients were hospitalized. The in-hospital mortality was 22 %, and the rate of re-admission at three months was 42%. The one year mortality was 54 %. The prognosis of the two groups was similar (Fig. 1). The median length of stays was 11 (7–16) days in the cardiac asthma group versus 12 (7–21) days in the classical CPE group (NS). In a multivariate analysis, the only variable available in the ED significantly associated with cardiac asthma was a previous history of COPD, with an odds ratio of 3.26 [95% confident interval: 1.61–6.58].

Pulmonary function tests were performed in 67 patients, and the mean time from admission to PFTs was 6 days. In the two groups, PFTs suggested a restrictive ventilatory defect (normal FEV_1_/FVC ratio). Cardiac asthma patients experienced obstructive airflow disorders in smaller distal airways (table 2).

## Discussion

In our study, cardiac asthma represented one third of CHF in elderly patients. Despite a higher percentage of hypercapnic acidosis in the cardiac asthma group, patients presented similar short and long term prognoses. Cardiac asthma patients exhibited greater peripheral airflow obstruction as shown by the reduced forced expiratory flow-rates at low lung volumes (Table 2)

Multiple studies have investigated the effects of CHF on lung mechanics. Patients with chronic CHF and orthopnea have a considerable increase in airflow resistance upon adopting the supine posture associated with supine expiratory flow limitation [22,23]. Patients with chronic, predominantly nonvalvular CHF frequently exhibit a restrictive ventilatory defect, due to enlarged heart size, increased intrathoracic fluids, and impaired inspiratory muscle strength [23]. While a restrictive defect may be seen in patients with both chronic heart failure (HF) and acute CHF, significant airflow obstruction is more likely to occur in the latter. Airway obstruction can occur during CHF through various mechanisms and with various degrees of severity [11-13,23-25]. Indeed, CHF can mimic acute asthma, a circumstance commonly referred to as "cardiac asthma." Pulmonary function studies have demonstrated increased airway resistance or decreased forced expiratory flows in CHF. The existence of dysfunction in small airways in CHF is suggested by an increased closing volume. Studies have suggested that with severe left HF there was a significant narrowing of the large airways. Finally, some patients with HF, when challenged with methacholine, exhibit nonspecific bronchial hyperresponsiveness. However, the majority of these studies did not clearly distinguish smokers from nonsmokers, a history of thoracic surgery, or significant obesity. Obstructive changes tend to be mild and appear to be more prevalent during periods of acute decompensation of CHF. They tend to improve with diuresis presumably due to a reduction in extravascular lung water, and a general reduction in pulmonary and bronchial blood volumes [24]. There also may be an enhanced degree of airway reactivity that diminishes with diuresis. Small improvements in expiratory flows are observed with anticholinergic and β2-agonist drugs in patients with chronic heart failure. Thus, data about the best treatments for cardiac asthma (i.e. are bronchodilators useful ?) are still lacking [16,26-30].

Wheezing is a frequent physical examination abnormality, common with acute asthma, exacerbation of COPD, and cardiac asthma. In the latter, wheezing is integrated in the Boston criteria for CHF, a clinicoradiographic validated score [20]. The prevalence of cardiac asthma has not been specifically reported in studies of heart failure. Nevertheless, recent studies found the rate of wheezing to be 10-15% in non-elderly patients with HF [2,6,31,32]. We demonstrated a high rate of cardiac asthma (35%) in elderly patients. Our study suggests that emergency physicians should focus on past diagnosis of COPD in patients with CHF and cardiac asthma, especially in those presenting with hypercapnia.

The population in our study of acute heart failure in elderly patients seems similar to previous studies in term of past medial history and prognosis. The in-hospital mortality was 22 %, compared to a mortality ranging from 13% to 29 % in other studies [2-8]. The rate of admission at 3 months was 42% close to a rate of early rehospitalization from 29 to 47%, within 3 to 6 months of the initial discharge [1,7,8]. Both groups had a similar prognosis (Fig. 1). Indeed, one could assume that cardiac asthma had a better prognosis because auscultation revealed fewer crackles, which could suggest less alveolar edema [23]. However, patients with cardiac asthma had higher hypercapnic acidemia which is thought to be associated with worse prognosis [7]. In fact, the usual variables for assessing severity (respiratory rate, PaO_2_, median values of BNP, rate of non systolic heart failure) confirmed similar severity of CHF in both groups.

The method used in our study to diagnose CHF and COPD requires further comment. For the diagnosis of CHF there is no ideal standard. Thus, as in several previous studies, we used consensus diagnosis by experts to assess the final diagnosis of CHF supported with results of Doppler-echocardiography when available during hospitalization [3], and BNP and NT-proBNP levels performed blindly at admission in the emergency room when available [18]. As in most ED, Doppler-echocardiography was not immediately available. It was performed in 72% of elderly patients in our institution, and PFTs were performed in 67 (32%) patients, with a mean time from admission to PFT of 6 days. These investigations could not been obtained in all patients, and we could not report lung volumes measured by pletysmography.

## Limitations of our study

Usually, COPD is usually defined by spirometry: i.e. presence of a FEV_1_/FVC<70% [21,22]. In our study, we used a more clinical approach to assess previous medical history of COPD, including symptoms, clinical and radiographic results. Unfortunately, previous spirometry was not available and not reported in our study [9]. The FEV1/FVC% was almost normal in the cardiac asthma group, and we hypothesis that our COPD group included patients with chronic bronchitis or emphysema and mild COPD rather than severe COPD [22].

The only criteria listed for "cardiac asthma" was wheezing reported by the physician's exam in the ED. There was no objective criteria given to differentiate this from co-existing lung disease and could lead to misclassification of CHF as being the primary event. However, the inclusion of the NT-proBNP and BNP data soundly confirms that this was a comparison of two cohorts with CHF (i.e. the wheezing group indeed did have CHF as reflected by the similar levels of BNP and NT-prBNP). At the time of the study, we were aware that patients with CHF were undertreated (especially with nitrates) because only two thirds of patients in both groups received either diuretics or nitrates [3].

Since the method used to evaluate expiratory flow limitation in our study is questionable, we suggest that obstructive pattern has been underestimated [30,33]. This is supported by a recent study which suggested that the negative expiratory pressure method was independent of FEV1/FVC % the usual index of overt obstruction in most groups of elderly subjects. Furthermore, some elderly patient, mainly female, suffering from dyspnea without medical history or decrease in FEV1/FVC %, appeared to be more flow limited during tidal expiration than those. Unfortunately, negative expiratory pressure method is not associated with routine PFTs in our institution.

Measurement of lung volumes by pletysmography was rarely performed, therefore we did not report the results of lung volumes measurement (total lung capacity). Thus, we can only suggest, and not strongly confirm that a restrictive defect was present in patients with CHF.

In our study, PFTs were performed according to physicians' orders, because the routine care of the patient was not modified. The percentages of patients who had PFT were quite similar in those with (35%) and without (30%) wheezing at the admission, thus we do not think that it could have biased the results. However, we agree that some hypothesis or conclusions are based upon a small number of patients who had PFTs (only one third). Due to the methodology of this observational study, we did not demonstrate that cardiac asthma was formally associated with distal airway obstruction. The limited sample size of our population precluded us to perform a case control study to confirm this hypothesis.

There was a low prevalence of bronchodilators given to the wheezing patients. This is attributed to the fact that the medical care was decided by the physicians in charge, and that bronchodilators have not been demonstrated to to be surely effective in cardiac asthma [16,26-29]. Although, bronchodilators were given in only one third of patients with wheezing in the emergency room, patients with cardiac asthma still had peripheral airway obstruction, suggesting a more marked airflow reduction if PFTs had been performed at admission to the emergency room. However for ethical reasons, PFTs were not performed at admission in the emergency room. The PFTs could have been modified if the medical care of patient had varied, especially prescription of bronchodilators or larger doses of diuretics. Thus, our results can not be extrapoled to the earlier stage of exacerbation of CHF.

## Conclusion

Cardiac asthma defined as CHF associated with wheezing represents one third of CHF in elderly patients. Cardiac asthma patients were more hypercapnic and had greater peripheral airway obstruction. However, in these patients short and long term outcomes were not dissimilar from those of patients with classical CPE.

## Competing interests

The author(s) declare that they have no competing interests.

## Authors' contributions

PR conceived of and designed the study, performed analysis and interpretation of data, edited the manuscript, and gave final approval of the manuscript. SJ performed interpretation of data, drafted the manuscript, and gave final approval of the manuscript. SD, MHB performed analysis and interpretation of data and gave final approval of the manuscript. RA, BR, RI and MB revised the manuscript critically for important intellectual content, and gave final approval of the manuscript.

## Pre-publication history

The pre-publication history for this paper can be accessed here:



## Figures and Tables

**Table 1:** Main Characteristics of Patients with Cardiac Asthma and Classical CPE

**Characteristics**	**Classical CPE**	**Cardiac asthma**
	**N = 137**	**N = 75**
***Demographic data***		
Age (years)	82 ± 9	83 ± 8
Male	62 (45%)	40 (53%)
***Past medical history***		
Tobacco user > 20 pack/year	47 (34%)	44 (59%)*
Diabetes mellitus	32 (23%)	17 (23%)
Hypertension	125 (91%)	62 (83%)
COPD	22 (16%)	35 (47%)*
Asthma	8 (6%)	6 (8%)
Venous thrombo-embolic disease	7 (5%)	7 (9%)
Heart failure	57 (42%)	27 (36%)
Ischemic cardiac disease	57 (42%)	34 (45%)
Peripheral arterial disease	14 (10%)	18 (24%)*
Cerebrovascular disease	21 (15%)	14 (19%)
***Previous medications***		
Beta-blockers	27 (20 %)	17 (23%)
Diuretics	86 (63%)	48 (64%)
Nitrates	63 (46%)	24 (32%)
ACE inhibitors	64 (47%)	27 (36%)
Inhaled bronchodilators	19 (14%)	36 (48%)*
Home oxygen	3 (2%)	12 (16%)*
***Symptoms and physical exam at admission***		
Heart rate (/min)	93 ± 21	93 ± 21
Ventilatory rate (/min)	29 ± 6	29 ± 6
Systolic arterial pressure (mmHg)	144 ± 32	155 ± 30
Diastolic arterial pressure (mmHg)	78 ± 18	82 ± 20
Clinical signs of acute ventilatory failure	116 (85%)	58 (78%)
Crackles with pulmonary auscultation	121 (88%)	52 (69%)*
Signs of RV failure	93 (68%)	45 (60%)
***Arterial blood gas***		
pH	7.43 ± 0.06	7.38 ± 0.08*
pH < 7.35	11 (8%)	20 (27%)*
PaCO_2 _(mmHg)	41 ± 11	47 ± 15*
PaCO_2 _≥ 45 mmHg	31 (23%)	32 (42%)*
PaO_2 _(mmHg)	67 ± 25	67 ± 18
Bicarbonates (mmol/L)	26 ± 5	27 ± 6
***Biological analysis***		
Creatinine clearance (mL/min)	40 (30–58)	44 (31–57)
***Treatments received in the emergency room***		
Oxygen	133 (97%)	74 (99%)
Diuretics	89 (65%)	52 (69%)
Vasodilators	26 (19%)	19 (25%)
Nebulized β2-Agonist	1 (1%)	29 (39%)*
Nebulized Ipratropium Bromide	4 (3%)	19 (25%)*

Data are mean ± SD, median [interquartile 25–75%], or number of patients (percentage) or extremes. CPE: cardiogenic pulmonary edema; COPD: chronic obstructive pulmonary disease. ACE inhibitors denotes angiotensin-converting-enzyme inhibitors or angiotensin-receptor blockers; RV: right ventricular signs including jugular venous pressure or abdominal jugular reflux or lower-extremity edema; signs of acute ventilatory failure included: orthopnea, abdominal respiration, use of accessory muscles; PaO_2_: partial oxygen pressure; SaO_2_: arterial oxygen saturation; PaCO_2_: partial carbon dioxide pressure; SpO_2_: peripheral oxygen saturation; Creatinine clearance was estimated using the Cockcroft formula; nebulized β2-agonists included terbutaline or salbutamol administration. *p < 0.05.

**Table 2:** Comparison of lung function parameters according to clinical presentation.

	**Classical CPE**	**Cardiac asthma**
	**N = 41**	**N = 26**
**FVC (L)**	1.77 ± 0.56	1.60 ± 0.73
**FVC (% predicted)**	67 ± 24	65 ± 26
**FEV**_1_**(L)**	1.33 ± 0,5	1.09 ± 0,49*
**FEV**_1_**(% predicted)**	65 ± 23	58 ± 27
**FEV**_1_**/FVC ratio (%)**	71.2 ± 15.6	66.9 ± 14
**FEV**_1_**/FVC (% predicted)**	96 ± 21	90 ± 21
**PEF (L/min)**	172 (119 – 238)	146 (100 – 206)
**PEF (% predicted)**	47 (31 – 64)	40 (30 – 55)
**FEF_25–75 _(L/s)**	0.99 (0.68–1.77)	0.76 (0.38–1.10)*
**FEF_25–75 _(% predicted)**	48 (24–73)	32 (18–51)*

Data are mean ± SD, median (interquartile 25–75%); FVC: forced vital capacity, FEV: Forced Expiratory Volume In 1 Second, FEF_25–75_: forced expiratory flow at 25% to 75% of vital capacity; PEF rate: peak expiratory flow; *p < 0.05.
